# Anorectal malformation patients’ outcomes after definitive surgery using Krickenbeck classification: A cross-sectional study

**DOI:** 10.1016/j.heliyon.2020.e03435

**Published:** 2020-02-20

**Authors:** Firdian Makrufardi, Dewi Novitasari Arifin, Dwiki Afandy, Dicky Yulianda, Andi Dwihantoro, Gunadi

**Affiliations:** Pediatric Surgery Division, Department of Surgery, Faculty of Medicine, Public Health, and Nursing Universitas, Gadjah Mada/Dr. Sardjtio Hospital, Yogyakarta, 55281, Indonesia

**Keywords:** Gastrointestinal system, Digestive system, Anatomy, Surgery, Abdominal surgery, Anorectal malformation, Constipation, Krickenbeck classification, Soiling, Voluntary bowel movement

## Abstract

**Background:**

The survival of anorectal malformation (ARM) patients has been improved in the last 10 years because of the improvement in management of neonatal care and surgical approaches for ARM patients. Thus, the current management of ARM patients are focusing on the functional outcomes after definitive surgery. Here, we defined the type of ARM and assessed the functional outcomes, including voluntary bowel movement (VBM), soiling, and constipation, in our patients following definitive surgery using Krickenbeck classification.

**Methods:**

We conducted a cross-sectional study to retrospectively review medical records of ARM patients who underwent a definitive surgery at Dr. Sardjito Hospital, Indonesia, from 2011 to 2016.

**Results:**

Forty-three ARM patients were ascertained in this study, of whom 30 males and 13 females. Most patients (83.7%) were normal birth weight. There were ARM without fistula (41.9%), followed by rectourethral fistula (25.5%), perineal fistula (18.6%), vestibular fistula (9.3%), and rectovesical fistula (4.7%). The VBM was achived in 53.5% patients, while the soiling and constipation rates were 11.6% and 9.3%, respectively. Interestingly, patients with normal birth weight showed higher frequency of VBM than those with low birth weight (OR = 9.4; 95% CI = 1.0–86.9; *p* = 0.04), while male patients also had better VBM than females (OR = 3.9; 95% CI = 1.0–15.6) which almost reached a significant level (*p* = 0.09). However, VBM was not affected by ARM type (*p* = 0.26). Furthermore, there were no significant associations between gender, birth weight, and ARM type with soiling and constipation, with *p*-values of 1.0, 1.0, and 0.87; and 0.57, 1.0, and 0.94, respectively.

**Conclusions:**

Functional outcomes of ARM patients in our hospital are considered relatively good with more than half of children showing VBM and only relatively few patients suffering from soiling and constipation. The frequency of VBM might be associated with birth weight and gender, but not ARM type, while the soiling and constipation did not appear to be correlated with birth weight, gender, nor ARM type. Further multicenter study is necessary to compare our findings with other centers.

## 1 Introduction

Anorectal malformation (ARM) is common congenital anomaly in newborns due to arrest of the caudal descent of the urorectal septum to the cloacal membrane. Its incidence is approximately 1 in 4,000–5,000 live births [1]. ARM can be classified according to the Krickenbeck classification [2].

The survival of ARM patients has been improved in the last 10 years because of the improvement in management of neonatal care and surgical approaches for ARM patients. Hence, the current management of ARM patients are focusing on the functional outcomes after definitive surgery [2, 3, 4, 5]. Several scoring system have been developed to evaluate these functional outcomes after surgery, however, they showed various findings [2, 3, 4, 5]. The Krickenbeck classification is developed to determine the ARM diagnostic classification system, operative procedure category and functional outcomes of ARM patients after surgery [6]. According to the Krickenbeck classification, the functional outcomes of ARM patients following definitive operation consist of voluntary bowel movement (VBM), soiling, and constipation [6]. Moreover, the Krickenbeck scoring system has been also widely used to assess the functional outcomes for children diagnosed with Hirschsprung disease after surgical procedures [7].

In this study, we aimed to: 1) determine the type of ARM, and 2) assess the functional outcomes in our patients following definitive surgery using Krickenbeck scoring system.

## 2 Results

### 2.1 Baseline characteristics

We analysed 43 medical records of ARM patients, consisting of 30 (69.8%) males and 13 (30.2%) females. Most patients (83.7%) were normal birth weight. Concerning the types of ARM among patients, there were most with no fistula (41.9%), followed by rectourethral fistula (25.5%), perineal fistula (18.6%), vestibular fistula (9.3%), and rectovesical fistula (4.7%) (Table 1).

**Table 1:** Baseline characteristics of 43 anorectal malformation patients after definitive surgery.

Characteristic	N (%)
Gender	
Male	30 (69.8)
Female	13 (30.2)
Birth weight	
Normal birth weight	36 (83.7)
Low birth weight	7 (16.3)
ARM type	
Perineal fistula	8 (18.6)
Rectourethral fistula	11 (25.5)
Rectovesical fistula	2 (4.7)
No fistula	18 (41.9)
Vestibular fistula	4 (9.3)

ARM, anorectal malformation.

### 2.2 Functional outcomes of ARM patients using Krickenbeck classification

The VBM was achived in 53.5% patients, while the soiling and constipation rates were 11.6% and 9.3%, respectively (Table 2).

**Table 2:** Functional outcomes of 43 anorectal malformation patients following definitive surgery according to Krickenbeck classification.

Functional outcome	N (%)
Voluntary Bowel Movement	
Yes	23 (53.5)
No	20 (46.5)
Soiling	
Yes	5 (11.6)
Grade 1	3 (7)
Grade 2	2 (4.6)
Grade 3	0
No	38 (88.4)
Constipation	
Yes	4 (9.3)
Grade 1	3 (7)
Grade 2	1 (2.3)
Grade 3	0
No	39 (90.7)

ARM, anorectal malformation.

### 2.3 Association between characteristics and functional outcomes of ARM patients

Interestingly, patients with normal birth weight showed higher frequency of VBM than those with low birth weight with odds ratio (OR) of 9.4 (95% confidence interval [CI]:1.0–86.9; *p* = 0.04), while male patients also had better VBM than females (OR = 3.9; 95% CI:1.0–15.6) which almost reached a significant level (*p* = 0.09). However, VBM was not associated with ARM type (*p* = 0.26) (Table 3).

**Table 3:** Association between characteristics and voluntary bowel movement, soiling and constipation in 43 anorectal malformation patients after definitive surgery.

Characteristics	N (%)	VBM		*p*	OR (95% CI)	Soiling		*p*	OR (95% CI)	Constipation		*p*	OR (95% CI)
		Yes	No			No	Yes			No	Yes		
Gender													
Male	30 (69.8)	19	11	0.09	3.9 (1.0–15.6)	26	4	1.0	1.8 (0.2–18.3)	28	2	0.57	0.4 (0.05–3.1)
Female	13 (30.2)	4	9			12	1			11	2		
Birth weight													
Normal birth weight	36 (83.7)	22	14	0.04*	9.4 (1.0–86.9)	32	4	1.0	0.8 (0.07–7.9)	33	3	1.0	0.5 (0.04–6.2)
Low birth weight	7 (16.3)	1	6			6	1			6	1		
ARM type													
Perineal fistula	8 (18.6)	4	4	0.26	N/A	7	1	0.87	N/A	7	1	0.94	N/A
Rectourethral fistula	11 (25.6)	8	3			9	2			10	1		
Rectovesical fistula	2 (4.7)	0	2			2	0			2	0		
No fistula	18 (41/9)	10	8			16	2			16	2		
Vestibular fistula	4 (9.3)	1	3			4	0			4	0		
ARM type													
Perineal fistula	8 (18.6)	4	4	1	0.8 (0.2–3.9)	7	1	1.0	1.1 (0.1–11.5)	7	1	1.0	1.5 (0.1–16.9)
Others	35 (81.4)	19	16			31	4			32	3		
ARM type													
Rectourethral fistula	11 (25.6)	8	3	0.18	3.0 (0.7–13.5)	9	2	0.59	2.1 (0.3–14.9)	10	1	1.0	1.0 (0.09–10.4)
Others	32 (74.4)	15	17			29	3			29	3		
ARM type													
Rectovesical fistula	2 (4.7)	0	2	0.21	6.4 (0.3–140.6)	2	0	1.0	1.3 (0.06–31.5)	2	1	1.0	1.7 (0.07–40.5)
Others	41 (95.3)	23	18			36	5			37	3		
ARM type													
No fistula	18 (41.9)	10	8	1.0	1.2 (0.3–3.9)	16	2	1.0	0.9 (0.1–6.1)	16	0	1.0	1.4 (0.2–11.3)
Others	25 (58.2)	13	12			22	3			23	4		
ARM type													
Vestibular fistula	4 (9.3)	1	3	0.32	0.3 (0.02–2.7)	4	0	1.0	0.7 (0.03–14.8)	4	2	1.0	0.9 (0.04–19.1)
Others	39 (90.7)	22	17			34	5			35	2		

*, significant (*p* < 0.05); ARM, anorectal malformation; CI, confidence interval; N/A, not applicable; OR, odds ratio; VBM, voluntary bowel movement.

Furthermore, there were no associations between gender, birth weight, and ARM type with soiling and constipation, with *p*-values of 1.0, 1.0, and 0.87; and 0.57, 1.0, and 0.94, respectively (Table 3).

## 3 Discussion

We are able to show patients with normal birth weight have a better VBM compared with those with low birth weight. VBM is affected by an adequate innervation and appropriate function of the pelvic floor, rectum, and anal spinchter. Low birth weight is associated with malnutrition [8]. These conditions might be correlated with the less adequate innervation and inappropriate function of the pelvic floor, rectum, and anal spinchter, resulting in the worse VBM in ARM infants with low birth weight compared with those with normal birth weight. In accordance with these findings, previous study also found that the improvements of the survival in ARM patients increased with birth weight [9]. Moreover, the innervation, pelvic floor, rectum, and anal spinchter in patients with ARM are not functioning properly due to anatomical anomalies or complications after reconstruction surgery [1]. Some pediatric surgeons with limited resources may perform a dilatation of perineal/vestibular fistula to increase the survival of ARM patients with very low birth weights [10].

The association between gender and VBM did not reach a significant level (Table 3; *p* = 0.09). This finding is consistent with previous study that also failed to reveal an association between gender and functional outcomes of ARM patients [11]. However, several hypothesis have been proposed to explain the difference of functional outcomes between male and female patients: 1) incorrect anoplasty (*i.e.* limited dissection of the rectum) in female children due to a fear of perforating the vagina; and 2) female patients less openly discussed with their families regarding their intestinal function, causing an intestinal management failure [12, 13].

Furthermore, we failed to find an association between ARM type and VBM. It was different from previous study that found the best functional outcomes were achieved in perineal fistula patients, while the worst findings happened in subjects with bladder neck fistula [14]. It was proposed that lower lesion of ARM shows better functional outcomes than higher lesion of ARM [1, 13]. The insignificant association between ARM type and VBM in our study might be related to the power of our study (0.71). These facts should be considered during the interpretation of our findings.

We also did not find any significant correlation between gender, birth weight, ARM type and soiling or constipation. Interestingly, patients with ARM lower lesion patients shows a higher possibility to have a constipation, whereas those with higher lesion revealed a higher risk to suffer a fecal incontinence [1]. There are several factors affecting the fecal continence, including sensation, voluntary muscle control and bowel motility [1]. Patients with lower lesion might have a continence as high as 90%, while those with higher lesion might reach a continence as low as 10% [15]. Previous report proposed some characteristics are good predictors for better outcomes in ARM patients, consisting of a normal anatomy of sacrum/spine, a good buttock crease and anal dimple, certain types of ARM, and absence of a sacral mass [13]. However, although the patients may have an ARM type with good prognosis, the incontinence and constipation are inevitable outcomes [16]. In addition, constipation might happen because of the continous process of dilatation in rectal pouch, resulting in inadequate peristaltis and failure of stool evacuation. Chronic constipation may lead to soiling due to overflow pseudoincontinence, in addition to defects of the sphincter muscle.

Most of our patients were males (69.8%) with normal birth weight (83.7%). It was compatible with previous reports, of whom males in 55–71% ARM cases [1, 10, 17, 18]. It is supposed that ARM patients often present with normal birth weight.

It should be noted that our study did not include other factors that might affect the functional outcomes of ARM patients after definitive surgery, such as associated anomalies, sacrum/spine anatomy, sacral ratio, surgical approaches, and complications [1, 13, 19].

Moreover, we suggest pediatric surgeons to apply the Krickenbeck classification during their practice to determine the type of ARM and the functional outcomes after surgery because it is a simple, practical and usable system. Krickenbeck classification also allows the different surgical procedures to be more comparable to each other.

## 4 Conclusions

Functional outcomes of ARM patients in our hospital are considered relatively good with more than half of children showing VBM and only relatively few patients suffering from soiling and constipation. Moreover, the frequency of VBM might be associated with birth weight and gender, but not ARM type, while the soiling and constipation did not appear to be correlated with birth weight, gender, nor ARM type. Further multicenter study is necessary to compare our findings with other centers.

## 5 Material and methods

### 5.1 Patients

In this cross-sectional study, we retrospectively evaluated the functional outcomes from the medical records of ARM patients who underwent a definitive surgery either in one-stage or three-stages at our hospital from June 2011 to June 2016, with minimum age of 3-year-old. Patients with incomplete data in their medical records and who underwent definitive surgery outside of our institution were excluded. The study was approved by the Institutional Review Board of the Faculty of Medicine, Public Health and Nursing, Universitas Gadjah Mada/Dr. Sardjito Hospital (KE/FK/1298/EC/2016). Parental consent was gathered from the patient investigated in this study.

### 5.2 ARM diagnosis and Krickenbeck classification

**Figure 1:**
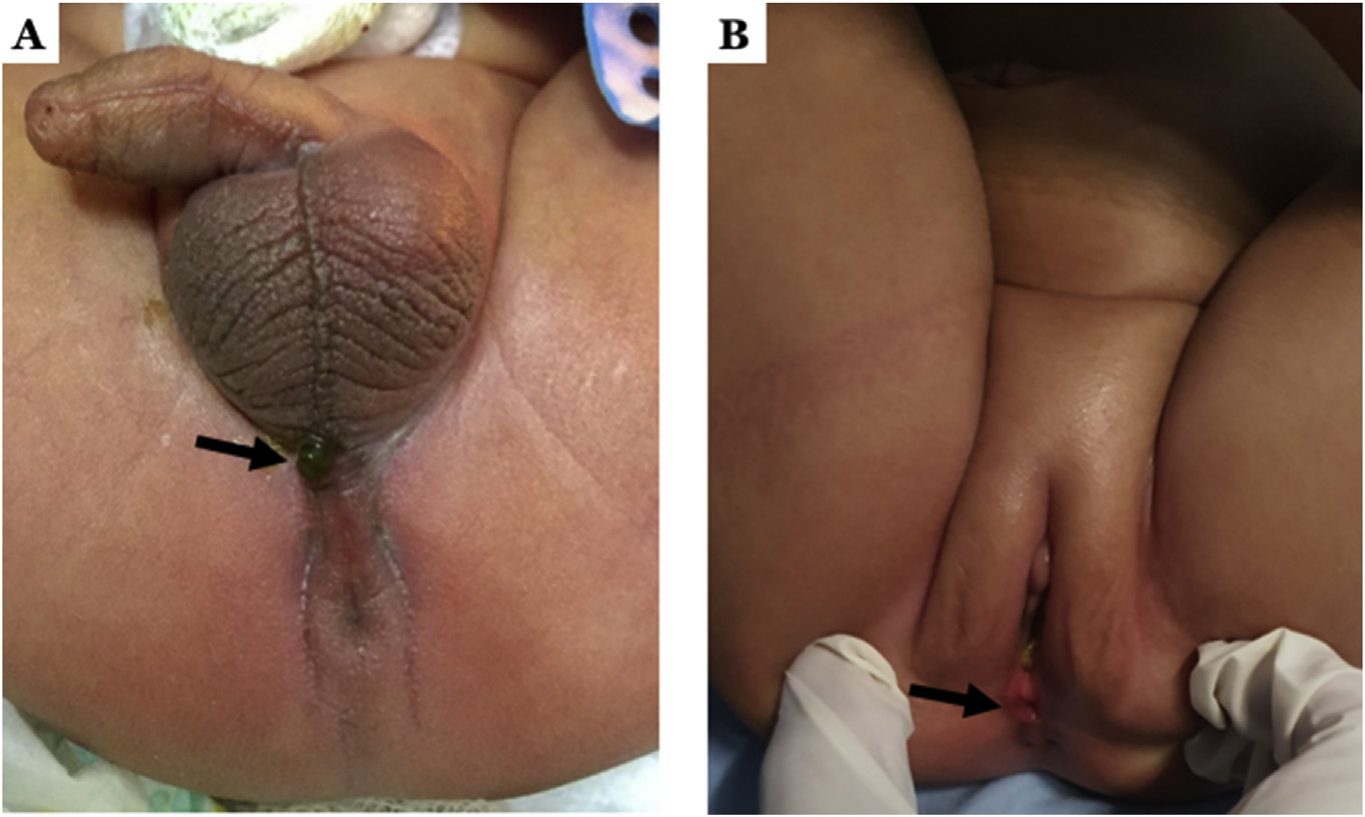
Anorectal malformation infant with (A) a perineal fistula (arrow); (B) an external vestibular opening of vestibular fistula (arrow).

Diagnosis of ARM was established according to clinical presentation and radiologic evaluation. First, we conducted a thorough perineal inspection when we saw a baby with an ARM. If meconium was visualized on the perineum, we diagnosed as a perineal fistula (Figure 1A), while if there was meconium in the urine, the diagnosis of a rectourinary fistula was established. In female infant, if there is an opening within the vestibule, we diagnosed as a vestibular fistula (Figure 1B).

**Figure 2:**
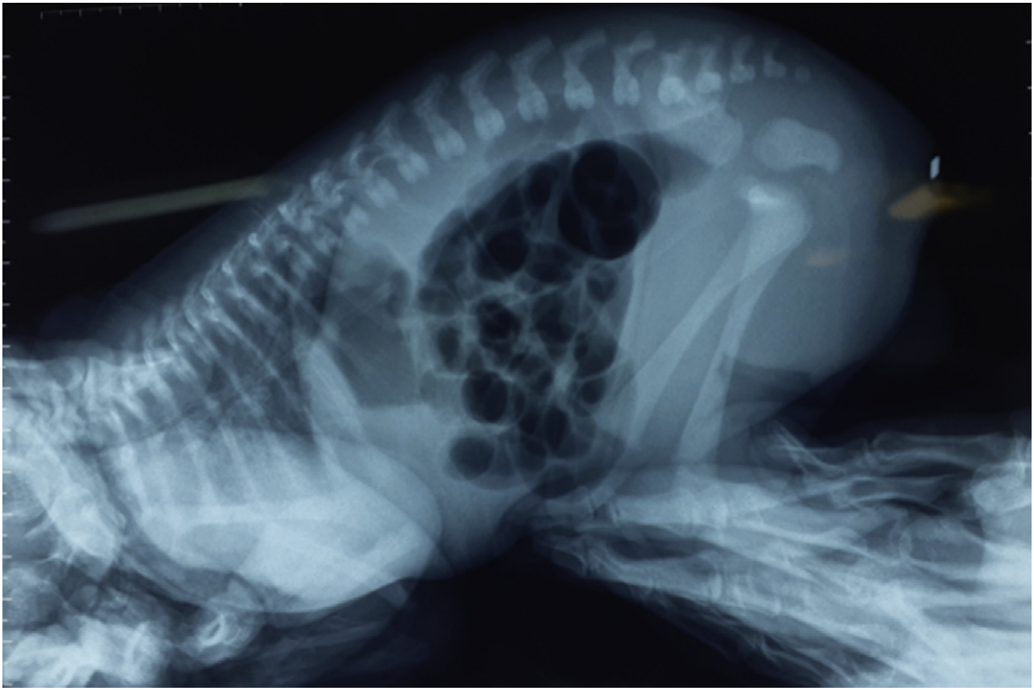
A plain cross-table lateral x-ray film with the newborn in prone position.

After 24 h, if no meconium is seen on the perineum or in the urine, we performed a plain cross-table lateral x-ray film with the newborn in prone position (Figure 2).

The types of ARM and functional outcomes after surgery were evaluated using the Krickenbeck classification [2, 3, 6, 7]. Krickenbeck classification determines the ARM types into two groups: 1) major clinical groups, and 2) rare/regional variants. Major clinical groups include perineal (cutaneus) fistula, rectourethral fistula (prostatic, bulbar), rectovesical fistula, vestibular fistula, cloaca, no fistula, and anal stenosis; while the rare/regional variants comprise pouch colon, rectal atresia/stenosis, rectovaginal fistula, H fistula, and others [6]. H fistula is an abnormal embryologic communication between anorectum and urethra without anal atresia [20]. According the Krickenbeck classification, functional outcomes after definitive surgery consist of: 1) VBM; 2) soling; and 3) constipation [6]. VBM is feeling an urge to defecate, the capacity to verbalize this feeling, and the ability to hold the bowel movement; while soiling consists of: a) grade 1, occasionally soiling (up to once or twice per week), b) grade 2, soiling every day but no social problems, and c) grade 3, constant soiling with social problems. In this classification, constipation includes: a) grade 1, manageable by changes in diet, b) grade 2, requires laxatives, and c) grade 3, resistant to laxatives and diet [6].

### 5.3 Statistical analysis

Data were presented as frequency (percentage). The associations between clinical characteristics and functional outcomes in ARM patients after surgery were determined using Fischer Exact test, with *p*-value of <0.05 considered as significant. By comparing the proportions of two independent samples, the estimated power of this study was 0.71. Odds ratios with their respective 95% confidence intervals were calculated to compare two independent groups on a dichotomous categorical outcome. All statistical analysis was done using the IBM Statistical Package for Social Science (SPSS) version 21 (IBM Corp., Chicago).

## Declarations

### Author contribution statement

F. Makrufardi: Conceived and designed the experiments; Performed the experiments; Analyzed and interpreted the data; Wrote the paper.

A. Dwihantoro: Conceived and designed the experiments; Wrote the paper.

Gunadi: Conceived and designed the experiments; Analyzed and interpreted the data; Wrote the paper.

D. Arifin, D. Afandy and D. Yulianda: Analyzed and interpreted the data; Wrote the paper.

### Funding statement

This research did not receive any specific grant from funding agencies in the public, commercial, or not-for-profit sectors.

### Competing interest statement

The authors declare no conflict of interest.

### Additional information

No additional information is available for this paper.
